# The association between the T309G polymorphism of the *MDM2* gene and sensitivity to anticancer drug is dependent on the p53 mutational status in cellular models

**DOI:** 10.1038/sj.bjc.6605096

**Published:** 2009-06-09

**Authors:** N Faur, L Araud, A Laroche-Clary, J Kanno, J Toutain, T Yamori, J Robert, V Le Morvan

**Affiliations:** 1 Département de Parmacologie, INSERM U916, Bordeaux, France; 2 Université Victor Segalen, Bordeaux, France; 3 Institut Bergonié, Laboratoire de Pharmacologie, 229 cours de l’Argonne, 33076 Bordeaux, France; 4 Division of Cellular and Molecular Toxicology, National Institute of Health Sciences, Setagaya-ku, Tokyo 158-8501, Japan; 5 Division of Molecular Pharmacology, Cancer Chemotherapy Center, Japanese Foundation for Cancer Research, Koto-ku, Tokyo 135-8550, Japan

**Keywords:** MDM2 polymorphism, p53 polymorphism, drug sensitivity, DNA-interfering drugs, NCI-60 panel, JFCR-45 panel

## Abstract

**Background:**

We investigated, in the panel of 60 human tumour cell lines of the National Cancer Institute (NCI-60), whether the R72P polymorphism of *TP53* and the T309G polymorphism of *MDM2* were associated to the *in vitro* cytotoxicity of anticancer agents, extracted from the NCI database. For validation, the same study was performed independently on a second panel of tumour cell lines, JFCR-45.

**Methods:**

Both SNPs were identified in cell DNA using PCR-RFLP techniques confirmed by direct sequencing and by pyrosequencing. For the analysis of the results, the mutational status of p53 was taken into account.

**Results:**

In the NCI-60 panel, the *TP53* rare-allele frequency was 32% and the *MDM2* rare-allele frequency 39%. The *MDM2* alleles were distributed according to Hardy–Weinberg equilibrium whereas this was only found, for the *TP53* alleles, in p53 non-mutated cell lines. Comparable results were obtained in the JFCR-45 validation set. The *TP53* SNP had low impact on anticancer drug cytotoxicity in either panel. In contrast, the *MDM2* gene polymorphism had a major impact on anticancer drug cytotoxicity, essentially in p53 non-mutated cell lines. Presence of the rare allele was associated to significantly higher MDM2 protein expression and to increased sensitivity to DNA-interfering drugs. In the JFCR-45 panel, a similar effect of the *MDM2* gene polymorphism was observed, but was less dependent on the p53 mutational status.

**Conclusions:**

We hypothesised that cell lines harbouring the *MDM2* G allele presented a lower availability of p53 for DNA repair, translating into higher sensitivity to DNA-damaging agents.

Numerous genetic factors influence the cytotoxicity of anticancer drugs and can explain, at least in part, the variability in individual responses to chemotherapy. Using *in vitro* models of the Centre d’Étude du Polymorphisme Humain, the group of ME Dolan at Chicago has evidenced the genetic components of drug response in non-tumour cells (Huang *et al*, 2007a, 2007b, 2008). We and others have used the NCI-60 model to show the relationships between the presence of a given polymorphic variation and the *in vitro* cytotoxicity of many anticancer drugs (Yarosh *et al*, 2005; Le Morvan *et al*, 2006; Moisan *et al*, 2006; Nief *et al*, 2007). The NCI-60 model consists of a panel of 60 human tumour cell lines of various origins which has served for the primary screening of thousands of potential anticancer drugs (Monks *et al*, 1991). The free availability of the databases elaborated by the Developmental Therapeutic Program (DTP) of the National Cancer Institute (NCI) allows to establish genotype/phenotype associations on a large number of drugs without performing drug cytotoxicity assays. The fact that the cell lines are of tumour origin adds a special advantage when the polymorphisms studied concern genes encoding drug targets or proteins involved in DNA repair, because the activity of such proteins in the tumour is expected to be important in drug cytotoxicity. On the contrary, polymorphisms in genes responsible for drug metabolism or transport would be more interesting to study in germ-line cells rather than in tumour cells, because they are expected to have a function in drug toxicity in the clinical setting.

The p53–MDM2 pathway is especially important for drug activity. Activation of p53 following exposure to cell stress leads to cell-cycle arrest and/or apoptosis and to DNA repair (Vogelstein *et al*, 2000). The loss of p53 function by oncogenic mutations provides cancer cells an opportunity to exacerbate their genetic instability and, hence, their tumourigenic and invasive properties (Lee *et al*, 1994). p53 is especially involved in response to DNA-damaging anticancer agents and *TP53* mutations lead to the loss of apoptosis induced by these agents (Lowe *et al*, 1994). Many studies have tried to relate the occurrence of tumour *TP53* mutations to drug activity with conflicting results (for reviews see Brown and Wouters, 1999; Gasco and Crook, 2003; Cimoli *et al*, 2004). For instance, when drug cytotoxicity is evaluated as inhibiting growth inhibition, cells bearing a *TP53* mutation are significantly less sensitive to a wide range of drugs than cells with wild-type *TP53*; however, when drug cytotoxic activity is evaluated as cloning efficiency inhibition, no difference appears between cells with mutated and wild-type *TP53* (Brown and Wouters, 1999). In the clinics, the question has not yet received definitive answers; it appears, however, that a loss of p53 function is frequently associated with resistance to treatment in several malignancies (Gasco and Crook, 2003). However, in a recent study on basal-like breast cancers treated by neo-adjuvant chemotherapy using an alkylating agent (cyclophosphamide), all tumours harbouring a mutated p53 presented a complete pathological response to treatment (Bertheau *et al*, 2007).

The *TP53* gene bears a polymorphism in exon 4, resulting in the replacement of an arginine residue by proline (R72P). This polymorphism has been found with an allele frequency of about 25% in Caucasian populations, giving rise to about 6% variant homozygous subjects. It has been shown that the R common form of p53 is able to induce apoptosis markedly better than does the P variant form in cell lines containing inducible wild-type p53 (Dumont *et al*, 2003). In *in vitro* models, the response to anticancer drugs such as cisplatin and doxorubicin appears higher for the R allele than for the P allele (Yarosh *et al*, 2005). The p53 polymorphic status has been determined in the NCI panel and has been associated with a decrease in drug cytotoxicity of alkylating agents in heterozygous cell lines (Sullivan *et al*, 2004) as compared to both variant and common homozygous cell lines. In the clinical setting, it has been shown in a series of advanced head and neck cancers that the polymorphism in wild-type p53 influences the clinical outcome of the treatment, with significantly shorter survival in homozygous patients with the P variant allele (Bergamaschi *et al*, 2003). In contrast, opposite results were obtained for patients with tumours harbouring a mutated form of p53: patients with the P allele had higher response rates than those expressing the R allele. Another example comes from the study of the response of patients with breast cancer to adjuvant tamoxifen; the p53 R72P polymorphism appears as a predictor of tamoxifen response, and the authors suggest that patients with breast cancer lacking the P allele might be candidates for other therapies (Wegman *et al*, 2006).

An important regulator of p53 activity is *MDM2*. Exploration of the p53 stress response pathway led to the discovery of a functional polymorphism in the *MDM2* intronic promoter (T309G, allele frequency around 30%) (Bond *et al*, 2004). The variant allele has been shown to have increased affinity to the *MDM2* transcriptional activator Sp1, resulting in higher levels of MDM2 protein and subsequent attenuation of the p53 pathway. This polymorphism is associated with accelerated tumour formation in both hereditary and sporadic cancers (for a review see Bond and Levine, 2007) and a meta-analysis recently concluded that variant homozygote G/G was associated with a significantly increased risk of all types of tumours (Hu *et al*, 2007). Its possible effect on anticancer drug response remains to be established, but a recent study comparing cell lines with different *MDM2* genotypes revealed a lower sensitivity of the G/G homozygous variant to topoisomerase II-interfering drugs (Nayak *et al*, 2007).

## Materials and methods

We used in this study two independent sets of human tumour cell lines for which *in vitro* sensitivity to a panel of anticancer drugs had been established, the NCI-60 collection (Monks *et al*, 1991) and the JFCR-45 collection (Nakatsu *et al*, 2005). DNA extracts from 59 of the 60 NCI cell lines of the panel were kindly provided by Dr S Holbeck, Cancer Therapeutic Branch, NCI, Bethesda, MD, USA. One cell line, MDA-N, is no longer available in the panel. DNA extracts were prepared from 42 cell lines of the JFCR-45 collection.

Polymerase chain reactions (PCRs) were performed on genomic DNA using appropriate primers (see below). Polymorphisms were detected in the NCI-60 panel by restriction fragment length polymorphism (RFLP) analysis on PCR products, using appropriate restriction enzymes. Electrophoresis of the PCR products was performed before and after digestion on 10% polyacrylamide gels. The presence of a variation was translated into the occurrence or the disappearance of a restriction site on the PCR product, leading to two shorter products. This technique allowed the unambiguous discrimination between homozygous cell lines with the common-allele, homozygous cell lines with the rare-allele and heterozygous cell lines, for both polymorphisms studied (G466C of *TP53*, rs1042522 and T309G of *MDM2*, rs2279744). Sequencing was performed on randomly chosen PCR products from the various genotypes of the variations studied. Concordance with RFLP was obtained in 100% of the cases.

We used the following primers for the G466C variation (R72P) of *TP53*: sense, 5′-TCCCCCTTGCCGTCCCAA-3′; antisense, 5′-CGTGCAAGTCACAGACTT-3′. The PCR products were then digested by *Bst*UI, which specifically cleaves the common G allele, and subjected to 10% polyacrylamide gel electrophoresis.

For the T309G variation of *MDM2*, we used the following primers: sense, 5′-GAGTTCAGGGTAAAGGTCAC-3′; antisense, 5′-TCAAGAGGAAAAGCTGAGTC-3′. The PCR products were digested by *Msp*AI, which specifically cleaves the variant G allele, and subjected to 10% polyacrylamide gel electrophoresis.

To confirm these results by a second independent technique, we also identified both polymorphisms by pyrosequencing. The DNA extracts of the JFCR-45 collection were exclusively studied by pyrosequencing. Direct sequencing of the PCR fragments without any further purification was performed on the Pyrosequencer PyroMark ID system (Biotage, Uppsala, Sweden) according to the instructions of the manufacturer. In that case, the primers we used were as follows: for *TP53*: sense, 5′ dR-Biotin-CACTGAAGACCCAGGTCCAGAT 3′; antisense, 5′-CCGGTGTAGGAGCTGCTGG-3′; *MDM2*: sense, 5′-CAGGGTAAAGGTCACGGG-3′; antisense, 5′ dR-Biotin-AGGCACCTGCGATCATCC 3′.

After identification of the genotypes of each cell line, the 50% growth-inhibiting concentrations (GIC_50_) of 136 core drugs vis-à-vis the 59 NCI-60 cell lines, expressed as –log_10_(GIC_50_), were extracted from the DTP database (http://dtp.nci.nih.gov). Similarly, the growth-inhibitory concentrations (GI_50_s) of 53 agents against the cell lines of the JFCR-45 collection were extracted from the original publication (Nakatsu *et al*, 2005). Drugs were grouped as a function of their known mechanism of action into eight categories (for details see Scherf *et al*, 2000): alkylating or platinating agents acting on N^7^ of guanine; other alkylating agents, acting on N^2^ and O^6^ of guanine; antimetabolites; antifolates; topoisomerase I inhibitors; topoisomerase II inhibitors; spindle poisons, subdivided into vinca-alkaloid-type and taxane-type mechanisms of action. With each cell line collection independently, it was possible to directly compare the mean GIC_50_ values of each drug in the various genotypes, and it was also possible to use a paired Student's *t*-test to analyse the data related to drug classes.

The mutational status of *TP53* of the NCI-60 collection was extracted from the NCI database, which integrates the data obtained by O’Connor *et al* (1997) and those obtained in the more recent study of Ikediobi *et al* (2006). There are some discrepancies between the two data sets and we chose the second one (Ikediobi *et al*, 2006) that results from genomic DNA resequencing. The JFCR cell lines had been also characterised for p53 mutational status by Frontier Science Co., Ltd (Ishikari, Hokkaido, Japan) according to the method described by Flaman *et al* (1995). The expression of *MDM2* in the NCI-60 collection was extracted from the DTP database. Numerous different data sets are available, 1 obtained by mRNA dot blots and 20 by Affymetrix microarray analysis. Unfortunately, there were no available data obtained by RT–PCR, which is considered as the reference method to quantify mRNA products. We chose in the database the most recent data set (September 2008 release), which indeed presented good correlations with most other data sets (Affymetrix U133A microarrays generated by Dr E Moler, ref. GC232415). *MDM2* expression was also obtained on Affymetrix microarrays in the JFCR-45 collection as described by Kanno *et al* (2006). Five *MDM2* probe sets were available on these arrays; based on expression intensity, we chose the probe 217373_x_at for comparisons of *MDM2* expression according to *TP53* and *MDM2* genotypes.

The *χ*^2^-test was used for comparing the distribution of the cell lines among genotypes. Pearson coefficients of correlation were computed for comparing continuous variables (GIC_50_ and gene expression data). To study the pharmacological parameters as a function of the genotype, we analysed the variances of drug GIC_50_s values or *MDM2* expression for each genotype and calculated the significance of the differences in mean values, using a general linear model taking into account the unbalanced size of the groups. The Bonferroni correction was applied to take into account the number of tests performed. The two cell line collections were studied independently, the NCI-60 collection as a training test and the JFCR-45 collection as a validation test, to decrease the probability of generating falsely positive observations.

## Results

### Identification of *TP53* and *MDM2* genotypes

**Figure 1:**
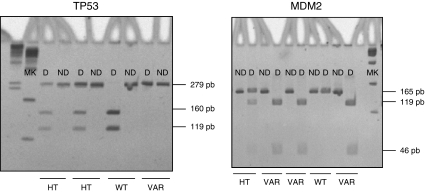
Representative electrophoretic patterns of PCR products before (ND) and after (D) digestion for the identification of *TP53* and *MDM2* polymorphisms. The deduced genotypes are indicated below each couple of lanes.

Figure 1 shows some representative electrophoretic profiles of PCR products before and after digestion with the appropriate restriction enzymes. Table 1 lists the 59 NCI cell lines and their genotypic status for the polymorphisms considered, as well as the p53 mutational status extracted from the NCI database. Some discrepancies between our results and those published earlier (Yarosh *et al*, 2005) were resolved by sequencing the complete exon 4, which confirmed the RFLP results. The results obtained by pyrosequencing were identical to those obtained by RFLP for all 59 cell lines. No special trend appeared concerning the presence or absence of a given genotype as a function of the tissue of origin of the cells. Concerning the *TP53* variation (Table 2), there were 35 cell lines with the homozygous common-allele genotype G/G, 10 heterozygous cell lines G/C and 14 with the homozygous rare-allele genotype C/C, giving a rare-allele frequency of 32% if all cell lines were diploid. This distribution was significantly different from the expected Hardy–Weinberg distribution (*P*<0.001). When considering separately the cell lines with wild-type and mutated p53, it appeared that the frequency of the rare C allele was significantly higher (*P*=0.026) in cell lines with mutated p53 (38.4%) than in cell lines with wild-type p53 (15.6%). Especially, all the cell lines with the homozygous C/C genotype were found among the p53-mutated cell lines. In addition, the heterozygous cell lines harboured less frequently a p53 mutation than homozygous cell lines. Concerning the *MDM2* variation (Table 2), there were 25 cell lines with the homozygous common-allele genotype T/T, 23 heterozygous cell lines T/G and 11 with the homozygous rare-allele genotype G/G, giving a rare-allele frequency of 38% and no significant distortion from a Hardy–Weinberg distribution. The same distribution was exhibited by cell lines with wild-type and mutated p53.

In the JFCR-45 panel, the rare *TP53* allele frequency was 25% and the proportion of heterozygous cell lines was also significantly lower than expected from Hardy–Weinberg distribution (Table 2). However, due to the lower number of cell lines, it was not possible to detect a significant difference between the rare-allele frequency in the p53 wild-type and the p53 mutated cell lines. As in the NCI panel, the heterozygous cell lines harboured less frequently a p53 mutation than homozygous cell lines. The rare *MDM2* allele frequency was 46% and the genotype distribution not significantly different from expected by the Hardy–Weinberg distribution (Table 2).

### Associations between genotypes and gene expression and drug cytotoxicity

**Figure 2:**
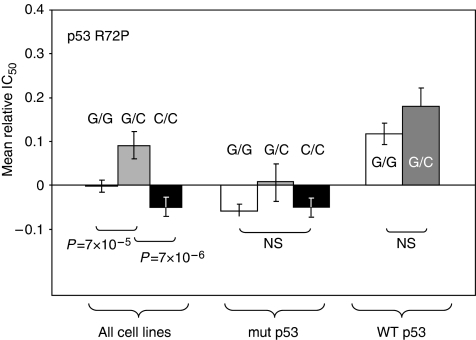
Schematic representation of the association between the *TP53* genotype of the cell lines of the NCI-60 panel and the cytotoxicity of alkylating agents. The GIC_50_s of the drugs of this class towards the 60 cell lines, expressed as −log_10_(GIC_50_), were normalised to zero, the cytotoxicities higher than the mean with a positive sign and the cytotoxicities lower than the mean a negative sign. Data are means±s.e.m. Cells were either taken all together (left) or as a function of the p53 status (middle and right). White columns, common homozygous cell lines; grey columns, heterozygous cell lines; black columns, variant homozygous cell lines.

**Figure 3:**
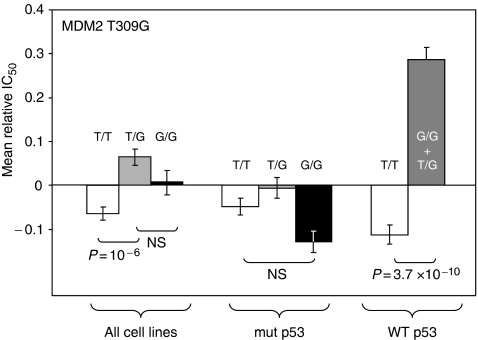
Schematic representation of the association between the *MDM2* genotypes of the cell lines of the NCI-60 panel and the cytotoxicity of alkylating agents. Same legend as Figure 2.

We looked for associations between the presence of a given genotype of *TP53* and the cytotoxicity of individual drugs of the NCI database. The statistical analysis took into account the fact that multiple comparisons were made. When the whole NCI-60 panel was considered, several significant associations could be found between the *TP53* variation and drug activity for some of the 136 drugs, such as fluorouracil, methotrexate, busulfan and cisplatin. The heterozygous cell lines appeared slightly more sensitive to these drugs than the rare-allele homozygous cell lines. Pooling the drugs as a function of their mechanism of action revealed that these differences were significant for alkylating agents (GIC_50_ ratio=1.4, *P*=7 × 10^−6^, Figure 2) and antifolates (GIC_50_ ratio=1.7, *P*=2 × 10^−5^, data not shown). However, these differences were not observed when the mutational status of p53 in the cell line panel was taken into account. Indeed, and independent of the polymorphisms of *TP53*, one can notice a highly significant difference between cell lines with wild-type and mutated p53: cell lines having a p53 mutation had a significantly lower sensitivity to most drugs than cell lines having no p53 mutation. The mean GIC_50_ ratio reaches 2 for alkylating agents (*P*=8 × 10^−8^, Figure 2) and 3 for antifolates (*P*=4 × 10^−4^, data not shown).

No consistent association between *MDM2* genotype and cell sensitivity to the 136 core drugs could be evidenced when they were sought in the whole NCI-60 panel. When the drugs were pooled as a function of their mechanism of action, a significant difference could be evidenced for alkylating agents: the cell lines containing a G allele were slightly more sensitive to this class of agents than the common homozygous cell lines (GIC_50_ ratio=1.3, *P*=4 × 10^−7^, Figure 3). However, when the cell lines were analysed separately as a function of the presence or absence of p53 mutations, it appeared that the polymorphism in the *MDM2* genes was associated with significant differences in drug cytotoxicity only in cell lines with wild-type functional p53, and not in cell lines harbouring a p53 mutation. In these cell lines, the presence of the rare G allele at position 309 of the *MDM2* gene was associated to an important and significant increase in the cytotoxicity of numerous individual DNA-interfering drugs (alkylating agents, inhibitors of topoisomerases I and II) (Table 3). When the drugs were pooled as a function of their mechanism of action, it appeared that the mean GIC_50_ ratio of alkylating agents in cell lines with no G allele *vs* cell lines with a G allele reached 2.5 for alkylating agents (*P*=3.7 × 10^−10^, Figure 3), 2.2 for topoisomerase I inhibitors (*P*=5.1 × 10^−7^, data not shown) and 2.4 for topoisomerase II inhibitors (*P*=3.8 × 10^−7^, data not shown). Due to the fact that only two cell lines had a homozygous G/G genotype in the p53 wild-type subset, it was not possible to study them separately from heterozygous cell lines, but it should be noticed that these two cell lines presented the lowest GIC_50_ values for most DNA-interfering drugs.

**Figure 4:**
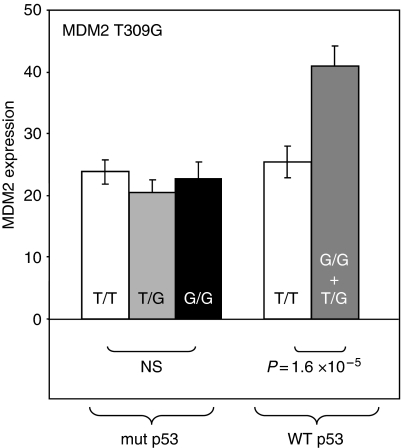
*MDM2* gene expression as a function of the genotype of the cell lines of the NCI-60 panel. *MDM2* expression was extracted from the DTP database (http://dtp.nci.nih.gov); the data set chosen originates from experiments on Affymetrix U133A microarrays realised by Dr E Moler, ref. GC232415. Cell lines with mutated p53 are on the left of the panel, and cell lines with wild-type p53 are on the right. White columns, common homozygous cell lines; grey columns, heterozygous cell lines; black columns, variant homozygous cell lines.

We also looked for associations between *MDM2* expression and polymorphism in the NCI-60 panel. As expected, there was a significantly lower *MDM2* expression in cell lines having a mutated p53 than in cell lines having no p53 mutation (expression ratio=1.57, *P*=1.5 × 10^−5^). There was no association between *MDM2* polymorphism and *MDM2* expression in the whole cell line panel; however, when the cell lines with and without a p53 mutation were considered separately, it appeared that, among the cell lines with no p53 mutation, those harbouring at least one G allele at position 309 of *MDM2* had a significantly increased *MDM2* expression (expression ratio=1.6, *P*=1.6 × 10^−5^) (Table 3 and Figure 4, right). This was not observed in p53-mutated cell lines (Figure 4, left). The association between *MDM2* expression and anticancer drug cytotoxicity was only weak, despite the fact that *MDM2* expression was higher in p53 wild-type cells, which are themselves more sensitive to DNA-damaging drugs. Especially, camptothecin and doxorubicin cytotoxicities were significantly correlated to *MDM2* expression in p53 wild-type cells.

In the JFCR-45 panel, we observed, as in the NCI-60 panel, a significant relationship between the mutational status of p53 and the cytotoxicity of most anticancer drugs belonging to the classes of alkylating agents, topoisomerase inhibitors and antimetabolites. When the cytotoxic drug panel was considered as a whole, the mean ratio of GIC_50_ values between wild-type and p53-mutated cell lines was 1.35 (*P*=6.0 × 10^−3^). However, the *TP53* polymorphism was not associated to differences in the cytotoxicity of any anticancer drug or drug class. The polymorphism of the *MDM2* gene was in contrast associated, as in the NCI-60 panel, to major differences in drug sensitivity, the cell lines harbouring the variant *MDM2* G allele being more chemosensitive than those harbouring only the common T allele; this was detected mainly in cell lines with wild-type p53 but also in cell lines where a mutation of p53 was detected, although to a lesser extent (Table 3). In p53 wild-type cell lines of the JFCR-45 panel, the mean GIC_50_ ratio of cytotoxic agents in cell lines with no G allele *vs* cell lines with a G allele reached 7.0 (*P*=2 × 10^−10^), whereas it was only 2.4 in p53-mutated cell lines. As in the NCI-60 panel, the expression of *MDM2* in the JFCR-45 panel was higher in p53 wild-type cell lines than in p53-mutated cell lines (expression ratio=2.0, *P*=2 × 10^−4^) and, in p53 wild-type cell lines, *MDM2* expression was higher in cell lines harbouring at least one G allele (expression ratio=1.9) (Table 3) but this did not reach significance (*P*=0.11) because of the small number of cell lines with common homozygous *MDM2* genotype.

## Discussion

The higher sensitivity of p53 wild-type cells, as compared to p53 mutated cells, to most anticancer agents (excluding spindle poisons), is a very general feature when cytotoxicity is evaluated by growth inhibition (Brown and Wouters, 1999). It was already observed by O’Connor *et al* (1997) in the NCI-60 panel and will not be further discussed here. The R72P polymorphism of p53 in the NCI panel had already been shown to be important in the cytotoxicity of several anticancer agents (Yarosh *et al*, 2005), especially those whose mechanism of action involves p53-mediated apoptosis. Yarosh *et al* (2005) had observed that the heterozygous cell lines were significantly more sensitive to alkylating agents than both common-allele and rare-allele homozygous cell lines. We found that this difference was significant only between heterozygous and rare-allele homozygous lines, but not between heterozygous and common-allele homozygous cell lines. The higher drug sensitivity of heterozygous cell lines in the NCI panel may be in fact related to the higher proportion of this genotype among the p53 non-mutated subset of the NCI-60 panel (31% *vs* 11% in the p53 mutated subset) and would not be a characteristic of the genotype. Indeed, there was no significant difference in drug sensitivity as a function of the *TP53* polymorphism in the JFCR-45 panel, which confirms that the difference seen in the NCI panel should be a bias due to the distribution of the genotypes. However, this last observation is interesting *per se*: the higher proportion of heterozygotes among wild-type p53 tumours in both panels has never been mentioned before. It could be simply related to the loss of heterozygosity at the *TP53* locus during evolution of p53 mutated, genetically unstable, tumours. It can also be hypothesised that the *TP53* heterozygous genotype may protect against the occurrence of p53 mutations. Researching both the p53 mutational status and the *TP53* polymorphism in clinical samples would be warranted to confirm this hypothesis.

The *MDM2* gene is one of the numerous transcriptional targets of p53 and the MDM2 protein induces the proteasomal degradation of p53. Its polymorphism present in the 5′ untranslated region has been consistently associated with an increased transcription rate and a subsequent attenuation of p53, leading to an activation of tumour formation and growth (Bond and Levine, 2007). The most striking observation made in our study is that, in both cell line panels studied independently, this polymorphism may be important in the cytotoxicity of anticancer agents against tumour cell lines, and that, in the NCI-60 panel, this effect is limited to the tumours having no p53 mutation. The cell lines harbouring at least one variant G allele of *MDM2* in this subset appeared 2- to 5-fold more sensitive to most DNA-interfering agents than the homozygous wild-type cell lines. This was not evidenced in cell lines with a mutated p53 and, therefore, should be related to the existence of a functional p53-dependent pathway. However, the higher drug sensitivity of the G allele-containing cell lines was observed in both p53 mutated and non-mutated cell lines of the JFCR-45 panel. This could be because not all p53 mutations exert the same effect on p53 function: it had been shown, for instance, that the 173H and the 273H mutations were able to induce apoptosis whereas most other mutations were not (Stähler and Roemer, 1998). A difference in the distribution of p53 mutations in the two panels could explain why the *MDM2* gene polymorphism is associated to chemosensitivity in the p53-mutated subset of the JFCR panel and not on the corresponding subset of the NCI-60 panel. In addition, the presence of the rare G allele in both cell line panels was associated with an increase in *MDM2* gene expression. This result was expected because the G allele of *MDM2* has increased affinity to the *MDM2* transcriptional activator Sp1.

The observation that the rare G allele of *MDM2* is associated with increased drug cytotoxicity in these two models was unexpected, because the reduction of p53 availability in the variant cell lines would be suspected to lead to a decrease in drug-induced apoptosis and cell death. In addition, it was shown in recent studies that this polymorphism was associated with a worse outcome of cancer disease (Gryshchenko *et al*, 2008). However, no clinical study has evaluated separately the overall prognosis of the disease and the predictive function of the polymorphism on response to treatment. The degree of malignancy of a tumour cell is related, at least in part, to its rate of proliferation, as is its sensitivity to antiproliferative agents. In experimental models as in the clinical setting, the most rapidly growing tumours are also the most sensitive to anticancer drugs. It has been shown that, for instance, in a series of 431 patients with breast cancer the most aggressive tumours (SBR grade III) responded better to neo-adjuvant chemotherapy than the less aggressive ones (SBR grade I) (*P*<10^−6^) (Amat *et al*, 2002). Consequently, the overall prognosis of a cancer is not simply related to its responsiveness to chemotherapy. In the NCI-60 panel, the cell doubling times have been evaluated and are significantly correlated with chemosensitivity; however, no relationship could be evidenced between the polymorphisms studied and the cell doubling times, showing that the association between *MDM2* polymorphism and drug cytotoxicity was independent from the rate of cell-cycle progression.

At the cellular level, it should be borne in mind that p53 is involved in many functions in the cell: it is not only in charge of inducing apoptosis in response to DNA damage (especially drug-induced DNA damage), but also of inducing DNA repair following drug-induced damage. One can hypothesise that the second function of p53 would be preponderant in the culture conditions of the cell line panels. As a consequence, the higher *MDM2* activity associated with the variant genotype would be responsible for a decrease in p53-mediated DNA repair, which would explain in turn why the cytotoxicity of DNA-interfering agents is higher in cell lines harbouring the variant G allele of *MDM2*. One can also hypothesise that the variant allele brings an additional alkylation site of for agents whose mechanism of action involves covalent binding to a guanine, which is the case of most alkylators; in these conditions, the cytotoxicity of these agents would be dependent upon the number of G alleles in the promoter of the *MDM2* gene. However, the higher drug sensitivity of T/G and G/G cell lines in comparison to T/T cell lines also applies to topoisomerase I-targeting and topoisomerase II-targeting drugs, rendering this mechanism unlikely.

## Figures and Tables

**Table 1:** Polymorphisms of the *TP53* and *MDM2* genes found in the NCI-60 panel

**Tumor type**	**Cell line**	**Mutational p53 status**	**SNP *TP53***	**SNP *MDM2***
Leukaemia	CCRF-CEM	Mut	G/C	T/G
		HL-60	Mut	C/C	T/G
		K-562	Mut	C/C	T/G
		MOLT-4	Mut	G/G	T/T
		RPMI-8226	Mut	C/C	T/T
		SR	WT	G/C	G/G
Lung cancer	A549/ATCC	WT	G/G	T/T	
		EKVX	Mut	G/C	T/G
		HOP-62	Mut	G/G	T/T
		HOP-92	Mut	C/C	T/T
		NCI-H226	WT	G/G	T/G
		NCI-H23	Mut	C/C	T/T
		NCI-H322M	Mut	G/G	G/G
		NCI-H460	WT	G/G	T/G
		NCI-H522	Mut	G/G	T/T
Colon cancer	COLO-205	Mut	G/G	T/T	
		HCC-2998	Mut	G/C	T/G
		HCT-116	WT	G/G	T/T
		HCT-15	Mut	G/G	T/G
		HT29	Mut	C/C	T/G
		KM12	Mut	G/C	G/G
		SW-620	Mut	G/G	T/G
Central nervous system	SF-268	Mut	G/G	T/G	
		SF-295	Mut	G/G	T/G
		SF-539	Mut	G/G	G/G
		SNB-19	Mut	G/G	T/T
		SNB-75	Mut	C/C	T/T
		U251	Mut	G/G	T/T
Melanoma	LOXIMVI	WT	G/G	T/G	
		MALME-3M	WT	G/G	T/G
		M14	Mut	G/G	G/G
		SK-MEL-2	Mut	C/C	T/T
		SK-MEL-28	Mut	C/C	T/T
		SK-MEL-5	WT	G/G	T/T
		UACC-257	WT	C/G	T/G
		UACC-62	WT	G/G	G/G
Ovarian cancer	IGROV1	Mut	G/G	T/T	
		OVCAR-3	Mut	G/G	T/T
		OVCAR-4	Mut	G/G	T/G
		OVCAR-5	WT	G/G	T/T
		OVCAR-8	Mut	G/G	T/T
		SK-OV-3	Mut	C/C	T/T
Renal cancer	786-0	Mut	G/C	T/G	
		A498	WT	G/G	T/T
		ACHN	WT	G/G	T/G
		CAKI-1	WT	G/C	T/G
		RXF-393	Mut	C/C	G/G
		SN-12C	Mut	G/G	G/G
		TK-10	Mut	G/G	T/G
		UO-31	WT	G/C	T/T
Prostate cancer	PC-3	Mut	C/C	T/T	
		DU-145	Mut	G/G	T/G
Breast cancer	MCF-7	WT	G/C	T/G	
		NCI/ADR-RES	Mut	G/G	T/T
		MDA-MB-231	Mut	G/G	T/G
		HS578T	Mut	G/G	G/G
		MDA-MB-435	Mut	G/G	G/G
		BT-549	Mut	C/C	T/T
		T-47D	Mut	C/C	G/G

**Table 2:** Distribution of *TP53* and *MDM2* genotypes between the NCI-60 and the JFCR-45 panels

**Polymorphism**	**Genotype**	**Whole panel**	**Wild-type p53**	**Mutated p53**
*NCI-60 panel*				
*TP53* R72P	G/G	35	11	24
		G/C	10	5	5
		C/C	14	0	14
*MDM2* T309G	T/T	25	6	19	
		T/G	23	8	15
		G/G	11	2	9
					
*JFCR-45 panel*					
*TP53* R72P	G/G	28	3	25	
		G/C	7	7	0
		C/C	7	3	4
*MDM2* T309G	T/T	15	4	11	
		T/G	15	6	9
		G/G	12	3	9

**Table 3:** *MDM2* expression and GIC_50_ values of three representative drugs against the NCI-60 and JFCR-45 cell lines as a function of the *MDM2* genotype and of the p53 mutational status

**p53 status**	***MDM2* genotype**	**Melphalan GIC_50_ (*μ*M)**	**Doxorubicin GIC_50_ (*μ*M)**	**Camptothecin GIC_50_ (*μ*M)**	**MDM2 expression**
*NCI-60 panel*					
Mutated	TT	31.6	0.140	0.044	23.8
	TG+GG	28.6	0.171	0.055	21.3
Wild type	TT	46.7	0.159	0.031	25.4
	TG+GG	13.3^a^	0.045^a^	0.016^a^	40.9^a^
					
*JFCR-45 panel*					
Mutated	TT	38.3	0.198	0.055	1.17
	TG+GG	25.7	0.099	0.014^a^	1.27
Wild type	TT	21.1	0.137	0.38	1.54
	TG+GG	16.3	0.055^a^	0.070^a^	2.92

a A significant difference according to the *MDM2* genotype.
